# Global phosphoproteomics of CCR5-tropic HIV-1 signaling reveals reprogramming of cellular protein production pathways and identifies p70-S6K1 and MK2 as HIV-responsive kinases required for optimal infection of CD4+ T cells

**DOI:** 10.1186/s12977-018-0423-4

**Published:** 2018-07-03

**Authors:** Danica D. Wiredja, Caroline O. Tabler, Daniela M. Schlatzer, Ming Li, Mark R. Chance, John C. Tilton

**Affiliations:** 1 Department of Nutrition, Center for Proteomics and Bioinformatics, School of Medicine, Case Western Reserve University, Cleveland, OH 44106 USA; 2 Department of Population and Quantitative Health Sciences, School of Medicine, Case Western Reserve University, Cleveland, OH 44106 USA

**Keywords:** HIV-1, CCR5, Signaling, Phosphoproteomics, Transcription, Splicing, Translation, Post-translational modifications

## Abstract

**Background:**

Viral reprogramming of host cells enhances replication and is initiated by viral interaction with the cell surface. Upon human immunodeficiency virus (HIV) binding to CD4+ T cells, a signal transduction cascade is initiated that reorganizes the actin cytoskeleton, activates transcription factors, and alters mRNA splicing pathways.

**Methods:**

We used a quantitative mass spectrometry-based phosphoproteomic approach to investigate signal transduction cascades initiated by CCR5-tropic HIV, which accounts for virtually all transmitted viruses and the vast majority of viruses worldwide.

**Results:**

CCR5-HIV signaling induced significant reprogramming of the actin cytoskeleton and mRNA splicing pathways, as previously described. In addition, CCR5-HIV signaling induced profound changes to the mRNA transcription, processing, translation, and post-translational modifications pathways, indicating that virtually every stage of protein production is affected. Furthermore, we identified two kinases regulated by CCR5-HIV signaling—p70-S6K1 (RPS6KB1) and MK2 (MAPKAPK2)—that were also required for optimal HIV infection of CD4+ T cells. These kinases regulate protein translation and cytoskeletal architecture, respectively, reinforcing the importance of these pathways in viral replication. Additionally, we found that blockade of CCR5 signaling by maraviroc had relatively modest effects on CCR5-HIV signaling, in agreement with reports that signaling by CCR5 is dispensable for HIV infection but in contrast to the critical effects of CXCR4 on cortical actin reorganization.

**Conclusions:**

These results demonstrate that CCR5-tropic HIV induces significant reprogramming of host CD4+ T cell protein production pathways and identifies two novel kinases induced upon viral binding to the cell surface that are critical for HIV replication in host cells.

**Electronic supplementary material:**

The online version of this article (10.1186/s12977-018-0423-4) contains supplementary material, which is available to authorized users.

## Background

HIV-1 enters cells via the interactions of gp120 with its primary receptor, CD4, and a coreceptor, typically CCR5 or CXCR4 for most clinical isolates. These events lead to conformational changes in gp120 that expose the fusion peptide of gp41 and lead to host and viral membrane fusion via the formation of an energetically favorable six-helix bundle conformation (reviewed in [1]). Transmission of HIV-1 is almost always associated with viruses that utilize CCR5 and these viruses predominate in the vast majority of infected individuals [2–7]. CXCR4-using viruses emerge in approximately 40% of patients infected with subtype B HIV [8, 9] and are associated with accelerated CD4+ T cell loss and progression to AIDS [2, 10, 11]. Emergence of CXCR4 is much less common in subtype C, which accounts for approximately half of infections worldwide [12–16].

CD4, CCR5, and CXCR4 are not only receptors and coreceptors for HIV-1 entry, but also have physiological roles in signal transduction in host immune cells. CD4 engagement results in the activation of the receptor tyrosine kinase p56^Lck^ while CCR5 and CXCR4 are G-protein coupled receptors (GPCRs) that link to Gα and Gβγ subunits, particularly Gα_i_, Gα_q_, and Gβγ (reviewed in [17]). HIV binding to the cell surface can induce signaling through CD4 and coreceptors and has been demonstrated to trigger calcium flux [18, 19] and to activate Pyk2 [20], phosphoinositol 3-kinase (PI3K) [19], Akt and Erk 1/2 [21], and the small GTPase Rac1 [22]. In addition, HIV can promote NF-κB, AP1, and NFAT translocation to the nucleus [23–25] and actin cytoskeleton rearrangement [21], including activation of cofilin, which reorganizes cortical actin barriers that restrict infection in primary CD4 T cells [26]. Rac1 and cofilin activation by HIV enhances viral entry into cells [22, 26], indicating that signaling through CD4 and coreceptor can induce rapid changes in cells that promote viral replication. However, signaling by HIV also can have longer-acting effects on the cellular environment: PI3 K activation by HIV was found to enhance infection via a post-entry step prior to integration [19] and, more recently, a phosphoproteomic screen demonstrated that CXCR4-tropic HIV causes extensive alterations to the cellular microenvironment including to the splicing factor SRm300 (SRRM2) that enhances viral production by regulating alternative splicing of HIV mRNAs [27].

Quantitative mass spectrometry-based phosphoproteomics is a powerful technique to broadly monitor phosphorylation changes in cells following exposure to a variety of stimuli. Here, we sought to build upon the study by Wojcechowskyj and colleagues [27] by performing a similar investigation but using CCR5-tropic HIV, which accounts for nearly all transmission events and the vast majority of infections. To gain additional insights, we examined phosphorylation changes at 1, 15, and 60 min after exposure to HIV and included conditions with the CCR5 antagonist maraviroc that blocks CCR5 signaling [28]. Together with an experimental setup closely related to that used by Wojchechowskyj, this design enabled comparisons between CCR5 and CXCR4-tropic HIV signaling, the transience or durability of phosphorylation changes, and how CCR5 signaling contributes to the overall reprogramming of host cells.

Here, we report that CCR5-tropic HIV signaling reprogrammed not only cellular mRNA splicing pathways, but also transcription, translation, and post-translational modification pathways. Signaling by HIV also induced rearrangement in phosphoproteins regulating cytoskeletal transport, endocytosis and movement of cargo along cytoskeletal networks. Surprisingly, many of these pathways were stimulated regardless of the presence of maraviroc, suggesting that the effects of HIV particles on cells are primarily due to CD4 signaling or from other host molecules incorporated into viruses rather than on the coreceptors themselves. These findings provide new insights into how HIV-1 actively manipulates the target cell environment to enhance its replication.

## Results

### Phosphoproteomics of CCR5-tropic HIV signaling in primary memory CD4+ T cells

**Fig. 1:**
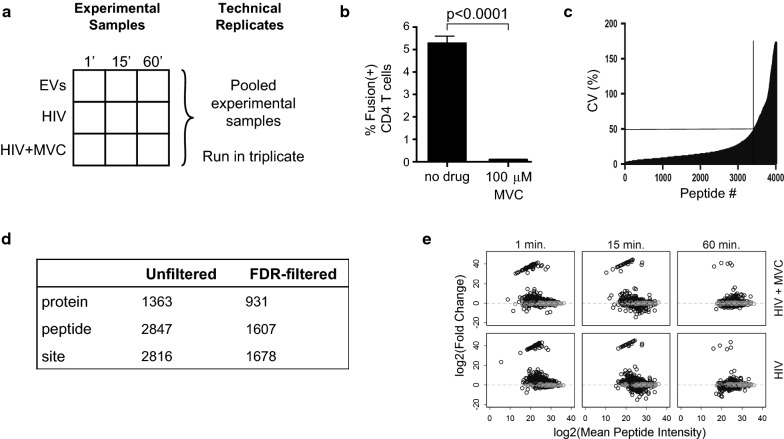
Phosphoproteomics of CCR5-tropic HIV signaling in primary memory CD4+ T cells. **a** Schematic of experimental design. Purified, unstimulated memory CD4+ T cells were exposed to extracellular vesicles (EVs), HIV, or HIV in the presence of 100 μm maraviroc (MVC) for 1, 15, or 60 min. A fraction of the experimental samples were pooled and run in triplicate for technical replicates. **b** Entry of HIV-1 THRO into primary CD4+ T cells is inhibited by treatment with 100 μM maraviroc. **c** Peptides demonstrating coefficients of variation (CVs) of > 50% in the technical replicate samples were excluded from further analysis. **d** Table of summary counts from the phosphoproteomics experiment; FDR-filtered results are selected if meeting the FDR = 0.001 cutoff. **e** Phosphopeptide-level MA plots across different treatment conditions and time points. Fold change (FC) calculations were in reference to extracellular vesicle control. Mean peptide intensity was calculated between the control and designated treatment condition. Grey circles indicate peptides that do not meet the log2(FC) cutoff

HIV-1 binding to the cell surface induces signals within CD4+ T cells that have been characterized primarily using biochemical approaches [18–26]. More recently, Wojcechowskyj and colleagues used a mass spectrometry-based proteomics approach to identify proteins that were differentially phosphorylated in primary resting CD4+ T cells following a 1-min exposure to CXCR4-tropic HIV [27]. CXCR4 is expressed on the majority of CD4+ T cells [29]; however, CXCR4-tropic HIV-1 variants account for only a fraction of viruses worldwide and almost none that are involved in transmission and early infection [2–7]. To gain insight into phosphorylation changes induced by CCR5-tropic HIV-1 binding to cell surface receptors, we collected leukapheresis samples from several donors and removed naïve CD4+ T cells—which express little CCR5 and are not infected to an appreciable extent by R5-tropic HIV—by rosette depletion to yield pooled, unstimulated memory CD4+ T cells. 150 × 10^6^ memory CD4+ T cells were exposed to 20 μg/ml p24 equivalent AT2-inactivated HIV-1 THRO or an equivalent protein concentration of non-viral extracellular vesicles. The high concentration of HIV was chosen to synchronize signaling events within CD4+ T cells as well as to maintain consistency with the CXCR4-tropic HIV phosphoproteomic study [27] to facilitate comparisons between R5- and X4-tropic HIV signaling. To further improve understanding of HIV-1 signaling events, we also exposed cells to R5-tropic HIV in the presence of 100 μM of the CCR5 antagonist maraviroc to block co-receptor signaling and included 1, 15, and 60 min stimulations for all experimental conditions to assess the durability of phosphorylation changes. A schematic of the experimental design is shown in Fig. 1a. Importantly, 100 μm maraviroc completely blocked entry of HIV-1 THRO into primary CD4+ T cells (Fig. 1b).

After applying filters to remove phosphopeptides demonstrating unreliable detection in three technical replicate controls (Fig. 1c), we removed peptides with non-phosphorylation post-translational modifications such as alkylation of cysteine residues. The final list contained 2816 phosphorylation sites from 1363 unique proteins using label-free proteomics. 1678 phosphorylation sites from 931 unique proteins were found to be HIV-responsive using an FDR cutoff of 0.001 (0.1%) compared to the respective extracellular vesicle control for at least one R5-tropic HIV-1 condition (Fig. 1d). The majority of peptides demonstrated modest fold-change variation (Fig. 1e); however a small number of peptides, particularly in the 1- and 15-min time points, showed much greater fold-change values Additional file 1: Table S1). We analyzed phosphopeptides with large fold-changes at both the 1- and 15-min time points and found nearly all were from proteins with roles in either (1) actin cytoskeletal architecture, endocytosis, and movement of vesicles along actin and tubulin networks, or (2) transcription, mRNA splicing and capping, translation, or acetylation of methionine residues on newly produced proteins. These data support previous studies demonstrating that these pathways are actively modulated by signaling upon HIV binding to the cell surface [22, 26, 27, 30].

### Signaling by CCR5-tropic HIV results in phosphorylation patterns with both significant overlap and differences compared to CXCR4-tropic HIV

**Fig. 2:**
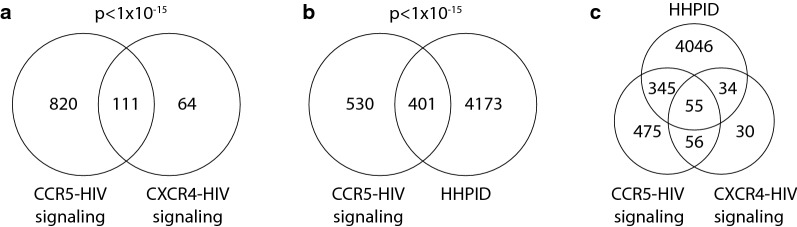
Venn diagram displaying overlap across three HIV datasets. CCR5-HIV signaling refers to HIV-responsive phosphoproteins (responsive in either HIV or HIV + MVC groups compared to MV controls) described in this study. CXCR4-HIV are HIV-responsive peptides reported by Wojcechowskyj and colleagues and HHPID is the HIV-1 Human Protein Interaction Database. There is statistically significant overlap of members between **a** the CCR5-HIV dataset vs. CXCR4-HIV (p < 1 × 10^−15^, hypergeometric test) and **b** between CCR5-HIV and the HHPID (p < 1 × 10^−15^, hypergeometric test). Both tests were calculated against a background of 21,764 protein-coding genes from the GeneCards database, as of March 2017. **c** Overlap of proteins in the CCR5-HIV, CXCR4-HIV, and HHPID datasets

HIV-1 binding to the cell surface can signal through several different pathways. First, specific interactions between gp120 and the host receptors CD4 and CCR5/CXCR4 can trigger responses in their respective signal transduction cascades. CD4 activates the receptor tyrosine kinase p56^Lck^ while CCR5 and CXCR4 signal via Gα and Gβγ subunits, particularly Gα_i_, Gα_q_, and Gβγ (reviewed in [17]). Second, signaling from gp120 interacting with non-receptor molecules has also been reported, such as the induction of cdc42 in dendritic cells via engagement of DC-SIGN [31]. It is possible that Env proteins could signal through other, currently unidentified cellular receptors as well. Third, HIV-1 incorporates host proteins into virions including HLA-DR, LFA-1, and ICAM-1 [32–34], which could also potentially induce signals in target cells. We reasoned that CCR5-tropic and CXCR4-tropic HIV would share considerable overlap in induced signaling pathways but could also vary due to potential signaling differences following CCR5 or CXCR4 engagement or due to the exclusion of naïve CD4+ T cells that are not readily infected by most HIV-1 isolates. Indeed, we found a considerable overlap between HIV-responsive proteins following CCR5- and CXCR4-HIV signaling (Fig. 2a), with 111 shared proteins between the two studies. Our study identified 63.4% (111/175, p < 1.0 × 10^−15^) of the HIV-responsive proteins identified by Wojecechowskyj and colleagues and an additional 820 HIV-responsive proteins not identified by their study. The larger number of HIV-responsive proteins found here is likely due to a combination of factors, including a larger number of total phosphopeptides detected, indicating more robust coverage of the CD4+ T cell proteome and a larger number of experimental conditions including 1, 15, and 60 min time points and CCR5-signaling blockade with maraviroc.

To gain further insight into the proteins identified as HIV-responsive following CCR5-HIV signaling, we compared our results with a manually curated list of human proteins shown to interact with HIV—the HIV-1 human protein interaction database (HHPID). This comparison was of particular interest as signaling has previously been demonstrated to influence host proteins levels that subsequently affect viral replication within host cells [19, 22, 26, 27]. Again, we found a highly significant overlap (Fig. 2b), with 43.1% (401/931, p < 1.0 × 10^−15^) of the HIV-responsive proteins identified here being part of the HHPID. The overlap between the HHPID, CCR5- and CXCR4-HIV signaling is shown in Fig. 2c. Shared proteins between the current study, CXCR4-HIV signaling, and the HHPID are listed in Additional file 1: Table S2.

### CCR5-tropic HIV signaling extensively reprograms cellular pathways involved in protein production and cytoskeletal regulation

Following integration into the host chromosome, HIV is transcribed into mRNA by the cellular RNA polymerase II complex. HIV contains at least four splice donors (D1–D4) and eight splice acceptors (A1–A8) that result in over 40 varieties of spliced mRNA transcripts. Splicing is regulated in a temporal fashion, with fully spliced 1- to 2-kb transcripts appearing first, followed by intermediate 4- to 5-kb transcripts, and finally by full-length 9-kb mRNAs that encode the Gag-Pol proteins and comprise the genome of budding viruses. The phosphoproteomic analysis of CXCR4-tropic HIV signaling performed by Wojcechowskyj and colleagues revealed a reprogramming of the cellular mRNA splicing pathways and identified SRRM2 as an HIV responsive protein that was also required for optimal HIV replication. Five additional SR proteins involved in mRNA splicing were identified as HIV responsive, including SRRM1, ACIN1, PNN, PPIG, and TRA1.

Our results with CCR5-tropic signaling strongly support the finding that HIV actively modulates splicing pathways: 5 of 6 of the SR proteins identified as CXCR4-HIV responsive were also CCR5-HIV responsive, the lone exception being PPIG. In addition, we identified a large number of additional splicing regulators including serine/arginine-rich splicing factors (SRSF) 1, 2, 5, 6, 7, 9, 10 and 11 and the upstream SRSF kinase 1. In particular, the SRSF1, 2, 5, and 6 proteins have previously been implicated in binding to the splice-donor region of the major 5′ splice site of HIV [35]. HIV reprogramming of mRNA splicing is also apparent on a more global scale: there was a highly significant enrichment of HIV-responsive proteins that were members of mRNA splicing and major mRNA splicing pathways in the REACTOME database (Additional file 1: Table S3).

In addition to the splicing pathways, bioinformatic analysis of the REACTOME databases revealed significant enrichment of HIV-responsive proteins in several other pathways relating to mRNA and protein production pathways: transcriptional regulation, processing of capped intron-containing pre-mRNA, metabolism of proteins, and post-translational protein modifications. These results suggest that HIV signaling induces profound changes at multiple levels of protein production, from mRNA transcription to post-translational modifications. In particular, the eukaryotic translation initiation (EIF) proteins, S ribosomal proteins (RPS), secretory (SEC) proteins involved in ER to Golgi transport, and the ubiquitin specific peptidase (USP) families all demonstrated multiple members that were responsive to HIV signaling.

Signaling by Rho GTPases and Rho GTPase cycle pathways were also highly enriched for proteins responsive to HIV signaling. Rho GTPases regulate a variety of cellular processes including cytoskeletal dynamics, transcription, endosomal trafficking, cell cycle, cell adhesion and cytokinesis. The observation that HIV signaling influences Rho GTPase signaling pathways has been explored by our lab recently, in combination with a small molecule screen that implicated Rho GTPases in regulation of HIV infection [30]. Briefly, CCR5-tropic signaling was found to dramatically alter the cellular Rho GTPase landscape, regulating not only cdc42 and Rac1 GTPases but also at least 25 guanosine dissociation inhibitors (GDIs), guanosine exchange factors (GEFs) and GTPase activating proteins (GAPs) that influence GTPase signaling. Furthermore, inhibition of cdc42, RhoA or Rho-associated protein kinase (ROCK) inhibited viral infection, demonstrating that the appropriate function of Rho family GTPases and their substrates is essential to optimal infection of CD4+ T cells. These results add to previous studies demonstrating that HIV-1 signaling actively regulates cytoskeletal dynamics and that inhibition of these pathways negatively affects viral replication [22, 26, 36].

### Identification of kinases activated or inhibited by CCR5-tropic HIV

**Fig. 3:**
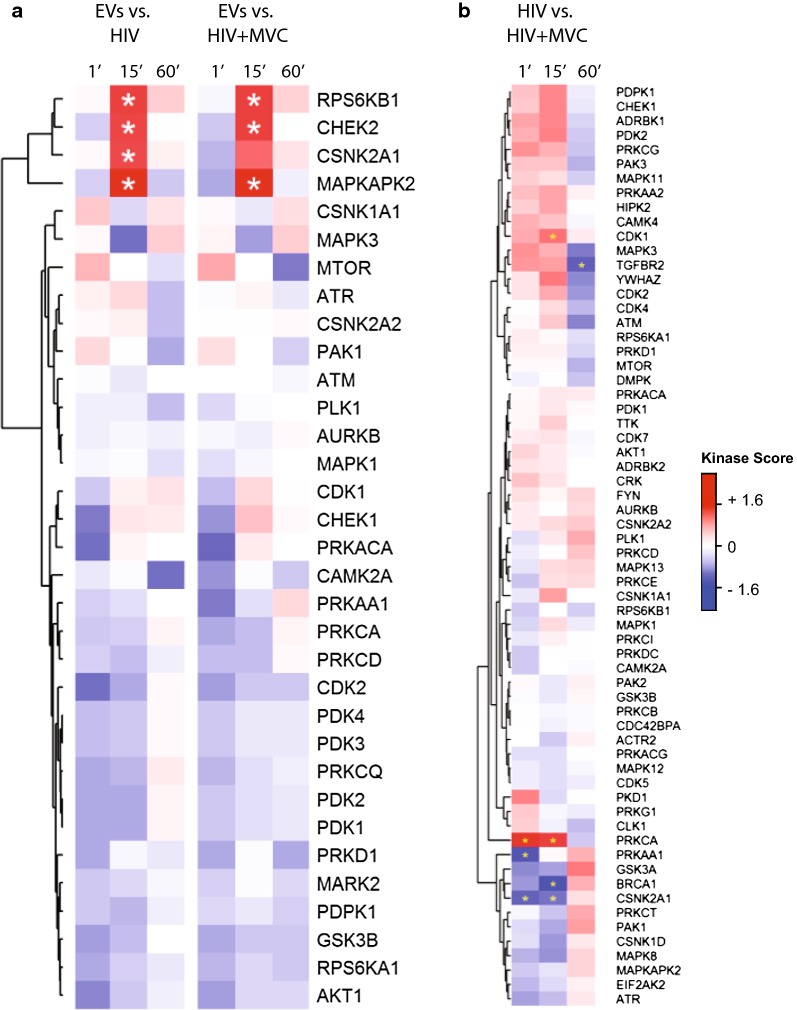
Kinase substrate enrichment analysis (KSEA) of HIV and HIV + MVC signaling. Only kinases with at least 3 identified substrates are included. Red colored cells indicate a positive normalized kinase score, which corresponds to a kinase with upregulated signaling output in the treatment group relative to control. The inverse (blue color) represents kinases with downregulated output. **a** Comparison between HIV or HIV + MVC with the extracellular vesicle (EV) control. **b** Comparison between HIV and HIV + MVC

One of primary advantages of phosphoproteomics is that it enables collection of information on thousands of differentially regulated phosphorylation sites that are the result of changes in the activity of proximal kinases and phosphatases acting upon their substrates. Since different kinases have varying preferences for consensus substrate motifs, a variety of algorithms to infer and identify cellular kinase activity based on phosphoproteomic data have been developed, including Scansite [37], Group-base Prediction System (GPS) [38], and kinase-substrate enrichment analysis (KSEA) [39]. Using the kinase to phosphosite annotations from KSEA, we identified four kinases with significantly upregulated activity 15 min after exposure to CCR5-tropic HIV: MAPKAPK2 (MK2), RPS6KB1 (p70-S6K1), CHEK2 (checkpoint kinase 2, CHK2) and CSNK2A1 (casein kinase 2 subunit a1) (Fig. 3a). CSNK2A1 kinase was not significantly upregulated when phosphopeptides from cultures containing maraviroc were analyzed. None of the analyzed kinases were significant at the 1- and 60-min time points, suggesting that in this study signaling transduction induced by HIV was most pronounced 15 min after exposure.

To gain further insight into the role of CCR5 signaling, we performed KSEA analysis comparing the HIV plus maraviroc condition to HIV alone, focused on the 15-min time point where kinase analysis was most robust. The CSNK2A1 kinase was significantly down regulated in HIV plus MVC compared to HIV alone (Fig. 3b), consistent with the loss of significance in the overall KSEA analysis. In addition, BRCA1 signaling was significantly reduced and PRKCA (protein kinase C, isoform alpha) and CDK1 (cyclin dependent kinase 1) activity were significantly upregulated when CCR5 signaling was inhibited. However, most kinases were not affected by maraviroc-mediated blockade of CCR5 signaling. Finally, we also performed cluster-guided pathway enrichment analysis comparing cells treated with HIV plus MVC compared to HIV alone to identify global cellular pathways affected by MVC in the presence of HIV. Proteins in the unfolded protein response (UPR), metabolism of proteins, signaling, and immune system pathways demonstrated significantly decreased phosphorylation in the presence of MVC (Additional file 1: Table S4).

### Phosphorylation changes induced by CCR5-tropic HIV-1 signaling are transient, but induce long-lasting effects required for optimal infection of primary CD4+ T cells

**Fig. 4:**
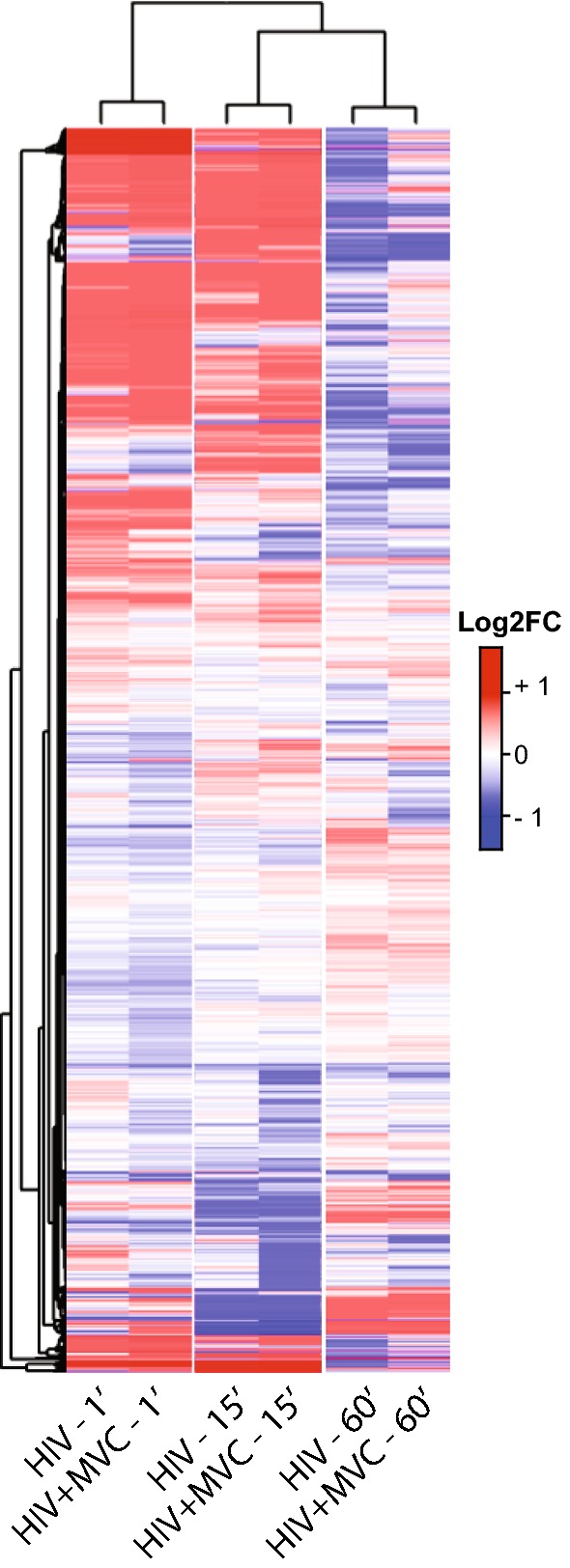
Phosphopeptide-level heat map of log2(FoldChange) peptide intensities calculated against vesicle control. Hierarchical clustering was applied to the rows and columns. All peptides with reliable detection (CVs ≤ 50%) are represented (no FDR filtering)

Although KSEA analysis did not reveal annotated kinases with significantly altered phosphopeptide levels at the 1- or 60-min time points, a large number of phosphopeptides (typically lacking annotations in such databases) were differentially regulated in these conditions (Fig. 4). Interestingly, we observed that many phosphopeptides that were up-regulated at the 1- and 15-min time points appeared to be down-regulated 60 min after exposure, and vice versa, suggesting that the immediate phosphorylation events appear to be quite short-lived and do not simply return to baseline, but rather show a reversal of phosphorylation status.

**Fig. 5:**
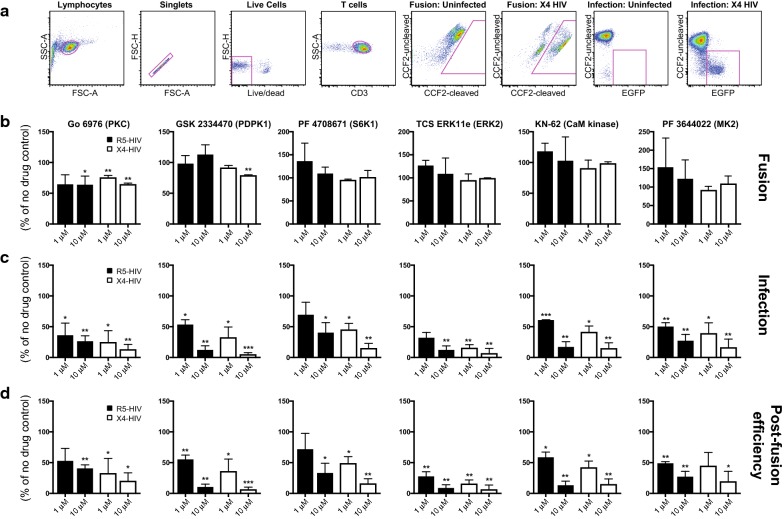
Effects of kinase inhibitors on HIV fusion and infection. **a** Gating strategy and representative fusion and infection plots in uninfected and infected primary CD4+ T cells. **b** Analysis of HIV fusion in the presence of PKC and PDPK1 inhibitors. Unstimulated CD4+ T cells were infected by combination reporter viruses pseudotyped with patient-derived CCR5- or CXCR4-tropic HIV Envs and bearing a β-lactamase-Vpr protein. The percentage of fusion represents the frequency of cells demonstrating cleavage of the CCF2 dye by flow cytometry 24 h after infection, normalized to no-drug controls. **c** Analysis of HIV infection in the presence of kinase inhibitors. Unstimulated CD4+ T cells were infected as above and LTR-driven EGFP expression monitored by flow cytometry 72 h after infection, normalized to no drug controls. **d** Analysis of viral post-fusion efficiency, calculated by dividing the percentage of infected cells by the percentage of fusion-positive cells. All experiments represent duplicate infections of CD4+ T cells from 3 independent healthy control subjects. *= p ≤ 0.05; **= p ≤ 0.01; ***= p ≤ 0.001

To determine whether the effects of CCR5-HIV signaling are longer-lived, we examined the role of kinases identified by KSEA in further detail. Briefly, we generated combination reporter viruses that contain both a β-lactamase-Vpr fusion protein that can be used in conjunction with the fluorescent FRET substrate CCF2-AM to monitor fusion with target cells [40, 41] as well as an *egfp* reporter gene that is expressed if fusion, uncoating, reverse transcription, nuclear import, integration into the host chromosome, Tat-dependent transcription and Rev-dependent mRNA export, and translation all occur successfully [42]. We purified primary resting CD4+ T cells from three healthy controls and infected with HIV-1 combination reporter viruses pseudotyped with patient-derived CCR5- or CXCR4-tropic Envs in the presence or absence of inhibitors targeting the differentially regulated kinases MK2 (PF 3644022), p70-S6K1 (PF 4708671), CHEK2 (Chk2 inhibitor) and CSNK2A1 (TBCA). In addition, we also included inhibitors of PKC (Go 6976) and its upstream regulator PDPK1 (GSK 2334470), ERK2 (TCS ERK11e), cyclin-dependent kinase 2 (CAS 222035-13-4), and calmodulin kinase (KN-62) as controls, as several of these have previously been demonstrated to affect HIV entry or replication [22, 43, 44]. Pharmacological inhibition of kinases was chosen over siRNA knockdown as the latter is still quite inefficient in primary CD4+ cells and certain barriers to infection in primary cells—such as cortical actin—are not present in cell lines [26]. The gating strategy and representative fusion and infection plots are shown in Fig. 5a.

Two kinase inhibitors impacted HIV fusion with host cells: Go 6976, an inhibitor of PKC, and GSK 2334470, an inhibitor of PDPK1 (Fig. 5b, Additional file 1: Table S5). Both kinase inhibitors showed significant inhibition of CXCR4-tropic HIV-1 fusion, while only 10 μM Go 6976 significantly inhibited CCR5-tropic entry. Inhibition of PDPK1 did not appear to affect CCR5-tropic HIV fusion. Inhibitors of PKC have previously been shown to reduce HIV fusion by Harmon and Ratner [22], and PDPK1 is an upstream regulator of PKC activity [45, 46], supporting the importance of the PKC pathway in HIV infection. The difference in significance between CCR5- and CXCR4-tropic HIV may be due to the higher percentage of CD4+ T cells expressing CXCR4 [29], resulting in larger numbers of infected cells and reduced variability between conditions. Go 6976 and GSK 2334470 also reduced infection of CD4+ T cells by HIV as measured by EGFP accumulation (Fig. 5c). Finally, we determined the EGFP:fusion ratio, which represents the fraction of cells that progress to infection following fusion. Even after controlling for reduced fusion, infection was still significantly diminished by treatment with PKC and PDPK1 inhibitors (Additional file 1: Table S5, Fig. 5d), indicating that these kinases influence both fusion as well as post-entry stages of infection.

In addition to inhibitors of PKC and PDKP1, drugs targeting ERK2, calmodulin kinase, p70-S6K1, and MK2 all significantly reduced EGFP expression compared to no drug controls (Fig. 5c, Additional file 1: Table S5). Importantly, inhibition of HIV infection was not a result of cytotoxicity as only one condition reduced cellular viability below 90% of the control: 10 μM of the ERK2 inhibitor TCS ERK11e. 1 μM of TCS ERK11e reduced infection significantly for both CCR5-tropic and CXCR4-tropic viruses while having no effect on viability, indicating that ERK2 activity is indeed required for optimal infection of primary CD4+ T cells. Inhibitors of CHEK2, CSNK2A1, and CDK2 did not significantly reduce CCR5-tropic HIV-1 infection but inhibited CXCR4-tropic HIV-1 at one or more concentrations (Additional file 1: Table S5). The p70-S6K1 and MK2 kinases identified by KSEA and shown here to be required for optimal HIV-1 infection of primary CD4+ T cells are known regulators of protein translation and cytoskeletal reorganization, respectively. These findings provide further evidence that these pathways are actively modulated by HIV-1 signaling and promote viral replication.

## Discussion

It has long been recognized that viruses, including HIV, rely on manipulation of host cell machinery to complete their replication cycles. For instance, the accessory proteins Vif, Vpr, Vpu, and Nef facilitate viral replication by degrading or blocking the activity of cellular proteins including APOBEC3G [47], UNG2 [48], helicase-like transcription factor (HLTF) [49], tetherin [50], and serinc-3 and -5 [51, 52]. More recently, it has become apparent that HIV-1 manipulation of the host cell environment begins even earlier, with signals mediated through CD4 and coreceptor facilitating fusion by activating Rac1 [22] and cofilin [26] as well as promoting post-entry stages of replication via PI3 K [19] or the splicing factor SRm300 (SRRM2) [27]. The extent of remodeling of the host cell was impressively demonstrated through an unbiased phosphoproteomic approach by Wojcechowskyj and colleagues and revealed over 100 proteins differentially regulated by signaling of a CXCR4-tropic HIV.

Here, we sought to build upon these pioneering studies by examining cellular alterations due to CCR5-tropic HIV, which accounts for the vast majority of HIV infections worldwide and nearly all transmitted viruses [2–7]. We anticipated that substantial overlap would exist in cellular proteins regulated by CXCR4- and CCR5-tropic signaling, as both viruses can bind CD4 and incorporate similar host molecules, including HLA-DR, LFA-1, and ICAM-1, that can also initiate signaling events. Indeed, we found a highly significant overlap between phosphorylated peptides in this study using R5-tropic HIV and the report by Wojcechowskyj and colleagues using CXCR4-tropic HIV. In particular, our data confirm and extend the observation that HIV signaling strongly influences mRNA splicing pathways. In addition to identifying five of six SR proteins identified in the CXCR4-HIV phosphoproteomic study, we identified 29 additional proteins differentially regulated by CCR5-tropic HIV that are members of the major mRNA splicing pathway in the REACTOME database. These results reveal that the reprogramming of the mRNA splicing machinery induced by HIV signaling is considerably more extensive than previously appreciated.

mRNA splicing activity is highly coordinated with—and sometimes coupled to—transcription. Indeed, many proteins involved in mRNA splicing have also been implicated in transcription, RNA 3′-end formation, nuclear export, and translation (reviewed in [53]). These processes were also dramatically affected by CCR5-tropic HIV signaling and, along with splicing, accounted for six of the ten most overrepresented pathways in the REACTOME database. The importance of translational machinery was further supported by the kinase substrate enrichment analysis (KSEA), which revealed that p70-S6K1 activity was upregulated by HIV-1 signaling, and by the reporter virus assays that demonstrated that inhibition of p70-S6K1 reduced infection of in primary CD4+ T cells. The p70-S6K1 protein is activated by mTOR and subsequently phosphorylates the ribosomal S6 protein (RPS6), eukaryotic transcription initiation factor 4B (EIF4B), and eukaryotic elongation factor 2-kinase (eEF2 k) and is a positive regulator of protein translation (reviewed in [54]).

Many of the proteins identified as responsive to HIV signaling are involved in multiple processes and dissecting the exact mechanisms will require careful experimental work. For instance, a recent report demonstrated that the lens epithelium-derived growth factor (LEDGF/p75), encoded by the *PSIP1* gene, interacts with splicing factors to target HIV integration to highly spliced genes [55]. LEDGF (PSIP1) and 33/60 of the LEDGF-interacting splicing proteins identified by Singh and colleagues [55] were identified here as CCR5-tropic HIV-responsive phosphoproteins, suggesting that they may have effects at the level of integration site selection as well as at mRNA splicing. Similarly, the p70-S6K1 is not only a critical regulator of transcription but also an inhibitor of autophagy, which reduces HIV-1 infection by selectively degrading Tat in primary CD4+ T cells [56]. Therefore HIV-1 signaling through p70-S6K1 could enhance viral replication both via enhanced protein translation and reduced Tat degradation.

One surprising result from our study was that inhibition of CCR5 signaling by maraviroc had relatively modest effects on HIV-responsive phosphoproteins. In contrast, previous studies have reported that CXCR4-tropic HIV signaling is required for cofilin activation and rearrangement of cortical actin barriers [26]. There are several possibilities for these apparently contradictory results. First, maraviroc has been demonstrated to block RANTES signaling through CCR5 [28], but it is possible that HIV Env interacts with the receptor in a different manner that is not fully suppressed by maraviroc. Second, CCR5 and CXCR4 may induce different signaling pathways in CD4+ T cells; for instance CXCR4 is highly expressed on naïve CD4+ T cells [29] that were excluded from this study. However, our results are in agreement with reports demonstrating that signal transduction via CCR5 is not required for viral replication [57–60].

## Conclusion

The results of this study confirm that HIV signaling has dramatic effects on cellular cytoskeletal regulation and on mRNA splicing pathways. We also demonstrate that CCR5-tropic HIV signaling induces significant reprogramming of virtually every stage of protein production: mRNA transcription, processing, translation, and post-translational protein modification. Furthermore, we identified two kinases not previously reported to have an effect on HIV replication—p70 S6K1 and MK2—that are induced by signaling and required for optimal infection of CD4+ T cells, providing new insights into how HIV manipulates host cells for viral replication.

## Methods

### Cells

This study was conducted according to the principles specified in the Declaration of Helsinki and under local ethical guidelines (Case Western Reserve University Institutional Review Board (IRB) #04-14-04). Normal donor samples were de-identified and obtained from leukapheresis from ALLCELLS, LLC. All donors were negative for HBV, HCV, and HIV. CD4+ T cells were isolated by adding additional autologous red blood cells to leukapheresis samples and RosetteSep CD4+ T cell enrichment kit antibodies (STEMCELL Technologies) prior to ficoll gradient separation. Cells were cryopreserved and treated with benzonase upon thawing, prior to infection.

### Phosphoproteomic analysis of HIV-exposed CD4+ T cells

Memory CD4+ T cells were purified by negative selection from a leukapheresis pack using custom RosetteSep kits (STEMCELL Technologies). Briefly, equal populations of 150 × 10^6^ cells were exposed to: (1) 20 μg/ml p24 equivalent AT2-inactivated HIV-1 THRO, (2) AT-2 inactivated THRO in the presence of 100 μM maraviroc, or (3) equivalent protein concentrations of extracellular vesicles, for 1, 15, or 60 min. HIV-1 THRO is a transmitted/founder virus isolated from a subject with early HIV infection that was found to be CCR5-tropic as evidenced by inhibition by TAK779 but not AMD3100 on TZM-bl cells and by its inability to replicate in PBMCs from a patient with the delta32-ccr5 mutation [61]. The AT2-inactivated viruses were derived from a cell clone of THRO (THRO CL.29) that was produced by co-culturing HEK293T cells transfected with the patient-derived THRO with A66-R5 cells and was identical in amino acid to the parental virus with the exception of Vif T68I, Vpu A8V, and Env R298K mutations. THRO CL.29 productively infected A66 cells expressing CCR5 but did not infect A66 cells that expressed CXCR4 (Julian Bess, personal communication). Extracellular vesicles were obtained as for the AT2-inactivated viruses with the exception that the HEK293T cells were not transfected with plasmids encoding THRO CL.29. Following exposure to virus or extracellular vesicles, cells were incubated with cold PBS containing protease and phosphatase inhibitors and washed and lysed with 2% SDS. Detergent removal was performed using the FASP cleaning procedure [62] and four hundred micrograms of each sample was digested enzymatically with a two step LysC/trypsin digestion. Phosphopeptides were enriched with titanium dioxide and enriched samples were analyzed by LC–MS/MS using a UPLC system (NanoAcquity, Waters) that was interfaced to Orbitrap ProVelos Elite mass spectrometer (Thermo Fisher). Technical replicates were performed by pooling equivalent protein amounts from each of the nine experimental samples following the titanium dioxide enrichment and analyzing them in triplicate by LC–MS/MS. Fold changes across time and treatment were determined for individual phosphopeptides compared to the corresponding extracellular vesicle controls. Unprocessed phosphoproteomic data is included in Additional file 1: Table S6.

### Bioinformatic analyses

#### Statistics

Posterior probabilities for every peptide and subsequent false discovery rates (FDRs) were calculated per condition based on the mixture-model employed by Wojcechowskyj et al. Since each peptide has a total of 6 posterior probabilities (one per treatment condition), the max was chosen as the representative value for that peptide when filtering based on the 0.1% FDR cutoff. Details are provided in the supplemental R code, adapted from the one provided by Wojcechowskyj et al. The phosphoproteomic data filtered by coefficient of variation (CV) used for FDR calculations, along with the results of the FDR calculations, are included in Additional file 1: Table S7 and 8, respectively.

#### Pathway enrichment

Proteins containing at least one phosphopeptide meeting the FDR cutoff were analyzed for statistical overrepresentation of REACTOME pathways (version 58) using the PANTHER Gene List Analysis tool [63].

#### Kinase-substrate enrichment analysis (KSEA)

Kinase scoring was based on the method originally described by Casado et al. using the PhosphoSitePlus Kinase-Substrate dataset for substrate identification. In short, a kinase’s output, reflected in its normalized score, is calculated from the collective phosphorylation changes of its identified substrates. A negative score (normalized by a weighted z-score) represents a kinase that has predominantly dephosphorylated substrates in the treatment condition relative to control. This, in turn, corresponds to downregulated signaling output with treatment. The inverse is true for a positive score. Statistical assessment was performed as originally described and utilized the z-test to assess the probability of achieving a more extreme score than the one observed. The KSEA scores and dataset used for KSEA analysis are included in Additional file 1: Table S9 and S10, respectively.

### Infection experiments

1 × 10^6^ unstimulated primary CD4+ T cells were plated per well in a 96-well format and incubated for 4 h at 37 °C with media alone or media supplemented with 10, 100 nM, 1, or 10 μM of indicated kinase inhibitors. Parallel plates were prepared for analysis of viral fusion and LTR-driven EGFP expression as previously described [42]. Following incubation with kinase inhibitors, cells were infected with 5 ng p24 equivalents of HIV-1 reporter virus strain NL4-3-deltaE-EGFP (obtained through the NIH AIDS Research and Reference Reagent Program, Division of AIDS, NIAID, NIH: pNL4-3-delta-E-EGFP (Cat #11100) from Drs. Haili Zhang, Yan Zhou, and Robert Siliciano) bearing the CCR5-tropic Env REJO.D12.1972 [64] or the CXCR4-tropic Env JOTO.TA1.2247 [65] and a β-lactamase-Vpr fusion protein [40, 41]. Plates were spinoculated for 2 h at 1200 rpm and 25 °C. Following centrifugation, cells were incubated for 1 h at 37° C. Cells for viral fusion analysis were treated with CCF2-AM, washed, and incubated overnight at room temperature in the presence of probenecid. For EGFP expression, cells were incubated at 37 °C for a total of 72 h prior to staining and processing. The following kinase inhibitors were added to cells 4 h prior to addition of virus and spinoculation: Go 6976 and PF3644022 (R&D Systems), PF 4708671, TBCA, TCS ERK11e, and GSK 2334470 (TOCRIS), KN-62 and CAS 222035-13-4 (Santa Cruz Biotechnology), and Chk2 inhibitor II hydrate (Sigma Aldrich) at the specified concentrations.

### Flow cytometry

Cells were washed once with PBS containing 1% BSA and incubated with live/dead fixable yellow dead cell stain (Thermo Fisher), CD3 Brilliant Violet 650 (BioLegend), and CD4 Allophycocyanin (eBioscience) at 4 °C for 30 min. Cells were washed in PBS/BSA and fixed in PBS/BSA containing 1% paraformaldehyde prior to data acquisition. All samples were analyzed using an LSRII flow cytometer (Becton–Dickinson). A minimum of 50,000 events were collected per sample. FlowJo version 9.7.6 (TreeStar, Inc) was used for analysis. Flow cytometry plots were gated using fluorescence intensity. Summary data are presented as fusion (+) and infection (+) cells as a percentage of no drug controls. Post-infection efficiency represent the ratio of infection (+):fusion (+) cells, also expressed as a percentage of no drug controls. All summary data represent mean values with standard error of the mean unless stated otherwise. All differences with a *p* value of < 0.05 were considered statistically significant, correcting for multiple comparisons when appropriate. Statistical analyses were performed using student T-tests within GraphPad Prism v7.0.

## Additional file

**Additional file 1: Table S1.** Phosphoproteomics data for peptides with large fold-changes at the 1- and 15- minute time points. **Table S2.** Shared proteins identified in the current study, CXCR4-HIV signaling, and the HIV-1 human protein interaction database. **Table S3.** Bioinformatic analysis of REACTOME pathways enriched for HIV-responsive phosphoproteins. **Table S4.** Cluster pathway enrichment analysis of HIV-responsive phosphoproteins. **Table S5.** Effects of kinase inhibitors on HIV-1 fusion, infection, and post-entry efficiency in primary CD4+ T cells.**Table S6.** Unprocessed proteomics data. **Table S7.** Phosphoproteomics data filtered by coefficient of variation (CV). **Table S8.** Phosphoproteomics data processed for false discovery rate (FDR) calculations. **Table S9.** Kinase-substrate enrichment analysis (KSEA) scores from phosphoproteomics data. **Table S10.** Dataset used for kinase-substrate enrichment analysis (KSEA) analysis.
